# Current Status and Temporal Trend of Potentially Toxic Elements Pollution in Agricultural Soil in the Yangtze River Delta Region: A Meta-Analysis

**DOI:** 10.3390/ijerph18031033

**Published:** 2021-01-25

**Authors:** Shufeng She, Bifeng Hu, Xianglin Zhang, Shuai Shao, Yefeng Jiang, Lianqing Zhou, Zhou Shi

**Affiliations:** 1 Institute of Applied Remote Sensing and Information Technology, Zhejiang University, Hangzhou 310058, China; 21814120@zju.edu.cn (S.S.); hubifeng@zju.edu.cn (B.H.); zhangxianglin@zju.edu.cn (X.Z.); sshuai@zju.edu.cn (S.S.); jiangyefeng@zju.edu.cn (Y.J.); LianQing@zju.edu.cn (L.Z.); 2 Department of Land Resource Management, School of Tourism and Urban Management, Jiangxi University of Finance and Economics, Nanchang 330013, China; 3 Institute of Soil Science, French National Institute of Agriculture, INRAE, 45075 Orleans, France

**Keywords:** potentially toxic elements, agricultural soil, Yangtze River Delta, meta-analysis, spatial distribution, temporal trend

## Abstract

Potentially toxic elements (PTEs) pollution in the agricultural soil of China, especially in developed regions such as the Yangtze River Delta (YRD) in eastern China, has received increasing attention. However, there are few studies on the long-term assessment of soil pollution by PTEs over large regions. Therefore, in this study, a meta-analysis was conducted to evaluate the current state and temporal trend of PTEs pollution in the agricultural land of the Yangtze River Delta. Based on a review of 118 studies published between 1993 and 2020, the average concentrations of Cd, Hg, As, Pb, Cr, Cu, Zn, and Ni were found to be 0.25 mg kg^−1^, 0.14 mg kg^−1^, 8.14 mg kg^−1^, 32.32 mg kg^−1^, 68.84 mg kg^−1^, 32.58 mg kg^−1^, 92.35 mg kg^−1^, and 29.30 mg kg^−1^, respectively. Among these elements, only Cd and Hg showed significant accumulation compared with their background values. The eastern Yangtze River Delta showed a relatively high ecological risk due to intensive industrial activities. The contents of Cd, Pb, and Zn in soil showed an increasing trend from 1993 to 2000 and then showed a decreasing trend. The results obtained from this study will provide guidance for the prevention and control of soil pollution in the Yangtze River Delta.

## 1. Introduction

Agricultural soil is the basis of human production and social development [1]. Compared with the world average, China faces severe pressure on per capita farmland resources [2]. As of late, however, the shortage of land resources in China has been exacerbated as a result of pollution by potentially toxic elements (PTEs), which is caused primarily by industrial production and rapid urbanisation [3,4,5]. Therefore, it is of great significance to assess the soil environmental quality for the rational use of land resources and the protection of public health.

The results of a national survey conducted between 2005 and 2013 showed that approximately 16.1% of the collected soil samples exceeded the safety limits countrywide, thus facing severe problems of PTEs pollution, especially in developed areas, such as the Yangtze River Delta (YRD) region [6,7]. Although the general state of national soil pollution can be ascertained from this survey, it does not provide a comprehensive understanding of the elemental distribution and concentrations. In addition to this national investigation, various studies have been conducted regarding specific contamination situations in many fields of interest [8,9,10,11,12]. However, most of this research focuses only on local regions, which provide an insufficient representation of the overall status of large regions. Thus, it would be valuable to establish a method to evaluate large-scale PTEs pollution in soil.

The temporal trend of soil pollution research is significant for regional soil environmental risk management [13,14]. However, currently, there is a lack of large-scale and long-term soil pollution field monitoring networks. Pollution situations at varying times can be obtained via a retrospective analysis of previously published studies [15,16,17,18]. Using this long-term trend information, researchers can reasonably predict the development trend of regional soil PTEs pollution and provide a decision basis for soil pollution prevention and control [19,20,21]

A meta-analysis is an effective strategy used to synthesise the results of multiple studies in order to obtain the overall trend of the target subject [22]. This method has been widely applied in evidence-based medicine and has been certified to be useful with ecological data. For example, researchers [23] compared 193 studies to evaluate the temporal yield stability of different cropping systems using a meta-analysis. Guo et al. [24] discovered the effect of land-use changes on soil carbon stocks by reviewing 74 publications. Nevertheless, few studies have conducted a large-scale and long-term analysis of PTEs pollution in agricultural soil using a meta-analysis [25,26].

As one of the most economically active regions in China, the YRD creates nearly a quarter of China’s Gross economic product with less than 4% of its land area. The YRD is densely populated (with a population of 227 million), and a large number of chemical, pharmaceutical, printing and dyeing plants, etc. located here. Human activities have a huge impact on the soil environment, therefore, soil PTEs pollution in the YRD has attracted increasing attention [27,28,29]. Therefore, this study conducted a meta-analysis based on 118 published papers from 1993 to 2020 to evaluate the status and temporal trends of PTEs pollution in its agricultural soil. The main objectives of this study were to (1) assess the overall pollution status of eight PTEs (Cr, Pb, Hg, As, Cd, Zn, Cu, and Ni) in the agricultural soil of the YRD; (2) explore the spatial pattern of PTEs pollution in the agricultural soil of the YRD; and (3) investigate the temporal trends of metal contents and identify potential drivers.

## 2. Materials and methods

### 2.1. Literature Collection and Data Extraction

Peer-reviewed 118 publications between 1993 and 2020 were collected using the keywords ‘potentially toxic elements’ OR individual elements (Cd, Cr, Hg, Pb, As, Cu, Zn, and Ni) AND ‘farmland soil’ OR ‘agricultural soil’ AND ‘Yangtze River Delta’ OR individual provinces (Zhejiang, Shanghai, Jiangsu, and Anhui) in the Web of Science and China National Knowledge Infrastructure databases. These primary studies were further screened according to the following criteria: (1) only field experiments monitoring surface (0–20 cm) soil in the YRD farmland region were collected; (2) selected studies should record the number of sampling sites and the size of the research area; (3) to ensure the quality of documents, the preparation, and analysis of soil samples should refer to the standards of China environmental protection industry [30,31]; and (4) the mean, standard deviation, and range could be extracted directly from the graphs, tables, and text, or could be calculated from the primary studies.

The extraction values of each study contained (1) the basic information (title, published year, author, keyword, and journal); (2) the location (name and administrative code of province, city, and country); (3) emission source (according to the description of the environment around the study area and the main anthropogenic emissions mentioned in the article), wherein the areas were divided into ‘normal group’ (rural farmland) and “HS group” (mining and smelting areas or industrial areas) for further subgroup analysis; and (4) the summary statistics of the contaminations (mean, standard deviation, maximum and minimum element contents for Cd, Cr, Hg, Pb, As, Cu, Zn, and Ni). The extracted data are presented in Supplementary 2.

### 2.2. Heterogeneity Test and Sensitivity Analysis

Due to the varying experimental regions and analysis methods, there was bias and heterogeneity in the primary extracted data. To ensure that the results were statistically significant, the data were tested and classified before analysis and calculation. The I^2^ statistic was calculated to assess heterogeneity [32] because it represents the percentage of individual heterogeneity in the total heterogeneity according to the values of Q and df. df represents the degree of freedom (k–1), and Q follows the chi-square test. An I^2^ less than 50% showed that multiple similar studies had homogeneity, and the fixed effect model was chosen to calculate the fitting effect value [33]. An I^2^ more than 50% showed heterogeneity, and thus, further sensitivity analyses, removal of outliers, or subgroup analyses were required. In this study, Cook’s distance method was chosen to find and remove outliers [34].

### 2.3. Chemical Analysis

The literatures selected in this study were monitored according to the national standard method of China for soil heavy metal content [30]. According to the standard, the total concentration of Cd, Zn, Ni, Cu, and Pb of the soil samples should be acid-digested with HCl-HNO_3_-HF-HClO_4_ and then analysed by atomic absorption spectrometer. Different studies may use different proportions of these acids and few studies used HCl-HNO_3_-H_2_O_2_ in the digestion procedure. The total Hg and As determination recommended for using cold atomic fluorescence spectrophotometry with digesting by H_2_SO_4_-HNO_3_-KMnO4 in the standard, or digested by HCl-HNO_3_ with bathing in the hot water can also be recognised [31].

### 2.4. Calculation of Weighted Mean Concentration

Weight is a very important indicator in the process of meta-analysis, as it is used to calculate the average value. All the studies selected for analysis herein were conducted with field experiments, and several important indicators could be used as reference values for weight calculations, including research area and the number of sampling sites, which determine the degree of representativeness of the study for the whole region, and the variation of the measured value, which determines the reliability of the study. Therefore, the weight value calculation in this study can be obtained using the following:

(1) $$W_{i}=A_{i}\times \frac{N_{i}}{Sd_{i}},$$

where $W_{i}$ is the weight related to each individual observation, and $A_{i}$, $N_{i}$, and $Sd_{i}$ are the size of the research area, the number of soil samples, and the standard deviation of the PTEs in each study, respectively.

The fitting effect value referred to is

(2) $$C=C_{i}\times \frac{W_{i}}{\sum_{i=1}^{n}W_{i}},$$

where C is the weighted mean, and $C_{i}$ and $W_{i}$ represent the calculated mean concentration of the PTEs reported and weight in each study, respectively.

To solve the problem of several studies having high weights that affect the fitting mean, the natural logarithm of the weight in every study was calculated using Equation (3), and the weighted mean was recalculated with $W_{i}^{*}$ as follows:

(3) $$W_{i}^{*}=\mathrm{lg}\left(A_{i}\times N_{i}/Sd_{i}\right),$$

(4) $$C^{*}=C_{i}\times W_{i}^{*}/\sum_{i=1}^{n}W_{i}^{*},$$

where $W_{i}^{*}$ and $C^{*}$ are the logarithmically transformed weight and recalculated weighted mean, respectively, and $A_{i}$, $N_{i}$, $Sd_{i}$, and $C_{i}$ have the same meaning as in Equations (1) and (2).

### 2.5. Potential Ecological Risk Evaluation

The effect of PTEs pollution on the biological population and potential ecological risk is described using the potential ecological risk index (RI) [35], which can be calculated using the following:

(5) $$E_{r}^{i}=\sum_{i=1}^{n}T_{r}^{i}\times \frac{C_{i}}{S_{i}},$$

(6) $$RI=\sum_{i=1}^{n}E_{r}^{i},$$

where $E_{r}^{i}\;$ represents the potential ecological index for a single element, $T_{r}^{i}$ is a fixed value that represents the toxin response (Cd = 30, Cr = 2, Hg = 40, Pb = 5, As =10, Cu = 5, Zn = 1, and Ni = 5), and $C_{i}$ and $S_{i}$ are the calculated mean concentrations of PTEs reported and the threshold, respectively.

### 2.6. Data Analysis

Data extraction and conversion were conducted using Microsoft Excel 2019 (v.2019, Microsoft Corporation, Redmond, WA, USA). The spatial distribution of the PTEs contents in the YRD was determined using ArcGIS (v10.3, ESRI Inc., Redlands, CA, USA). A meta-analysis was performed using R (v.3.5.3, AT&T, Murray, NJ, USA) with the meta and metafor packages [36].

## 3. Results

### 3.1. Publication Bias and Outlier Analysis

**Figure 1:**
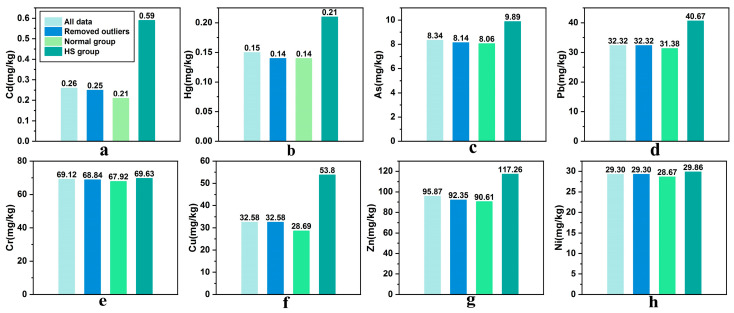
Weighted mean values of (**a**) Cd; (**b**) Hg; (**c**) As; (**d**) Pb; (**e**) Cr; (**f**) Cu; (**g**) Zn; and (**h**) Ni in different groups.

In the data analysis influenced by extreme values, the distribution of the eight elements’ concentrations represented various degrees of skewness (Figure S1, Supplementary 1). Thus, Cook’s distance method was used for outlier diagnosis. Meanwhile, we noticed that agricultural areas at higher risk of pollution are more likely to attract researcher attention, such as mining and smelting areas and industrial production areas, which might introduce extreme values. Therefore, a subgroup analysis was performed to identify publication bias, wherein the data were divided into ‘HS’ and ‘Normal’ groups according to their emission source. Figure 1 shows the comparison of the weighted average calculated by the four datasets, which were ‘all data’ (all data collected from the literature), ‘removed outliers’ (all data excluding outliers), ‘normal group’ (rural farmland), and ‘HS group’ (mining and smelting or industrial areas). We found that the mean values of the HS group were obviously higher than those of the normal group, especially for Cd, Hg, and Cu, which proved the existence of publication bias. However, there were no remarkable differences between the contents of removed outliers and normal group, suggesting that publication bias had no significant impact on the overall results. Therefore, the data with the outliers removed were used in the subsequent analyses.

### 3.2. Overall Status of Soil PTEs Content in the YRD

The overall regional PTEs contents in the YRD agricultural soil are summarised in Table 1. The sampling number showed that more attention was paid to Pb (175) and Cd (153), and less to Zn (104) and Ni (49). The regional mean concentrations of Cd, Hg, As, Pb, Cr, Cu, Zn, and Ni were 0.25 mg kg^−1^, 0.14 mg kg^−1^, 8.14 mg kg^−1^, 32.32 mg kg^−1^, 68.84 mg kg^−1^, 32.58 mg kg^−1^, 92.35 mg kg^−1^, and 29.30 mg kg^−1^, respectively. Compared with the national standard, the proportion of samples that exceeded the screening value (GB 15618-2018) [6] was the highest for Cd, wherein approximately 26.14% of the samples were contaminated by Cd, followed by Zn (6.94%) and Pb (3.43%). There were no samples with excessive Cr or Ni concentrations. Nevertheless, in general, the weighted mean concentration of the eight PTEs was within the safe range, exhibiting varying degrees of accumulation compared with the background values.

### 3.3. Spatial Distribution Pattern of Soil PTEs in the YRD

**Figure 2:**
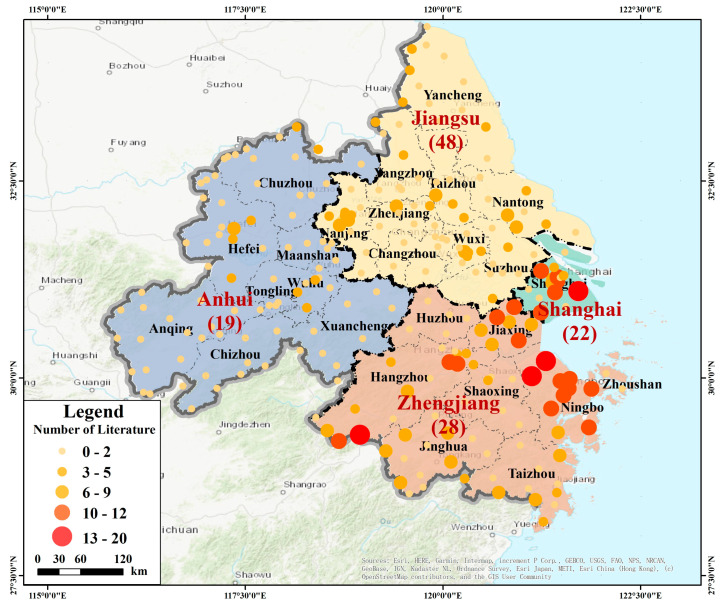
Literature quantity statistics of agricultural soil potentially toxic elements (PTEs) pollution in the Yangtze River Delta (YRD).

**Figure 3:**
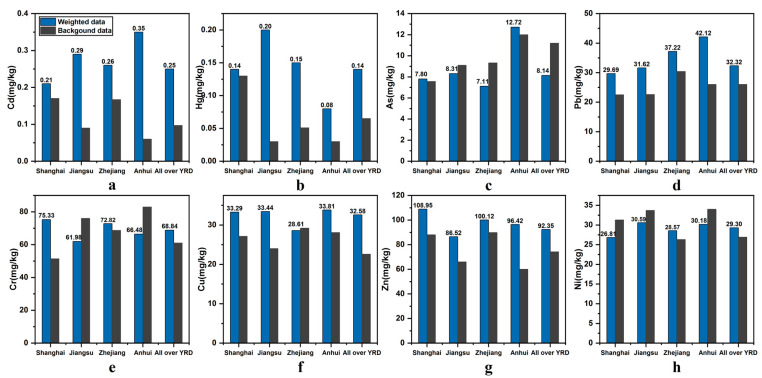
Average concentrations of (**a**) Cd; (**b**) Hg; (**c**) As; (**d**) Pb; (**e**) Cr; (**f**) Cu; (**g**) Zn; and (**h**) Ni in four provinces and the entire Yangtze River Delta (YRD) (background value reference: Shanghai [38], Jiangsu [39], Zhejiang [40], and Anhui [41]).

**Figure 4:**
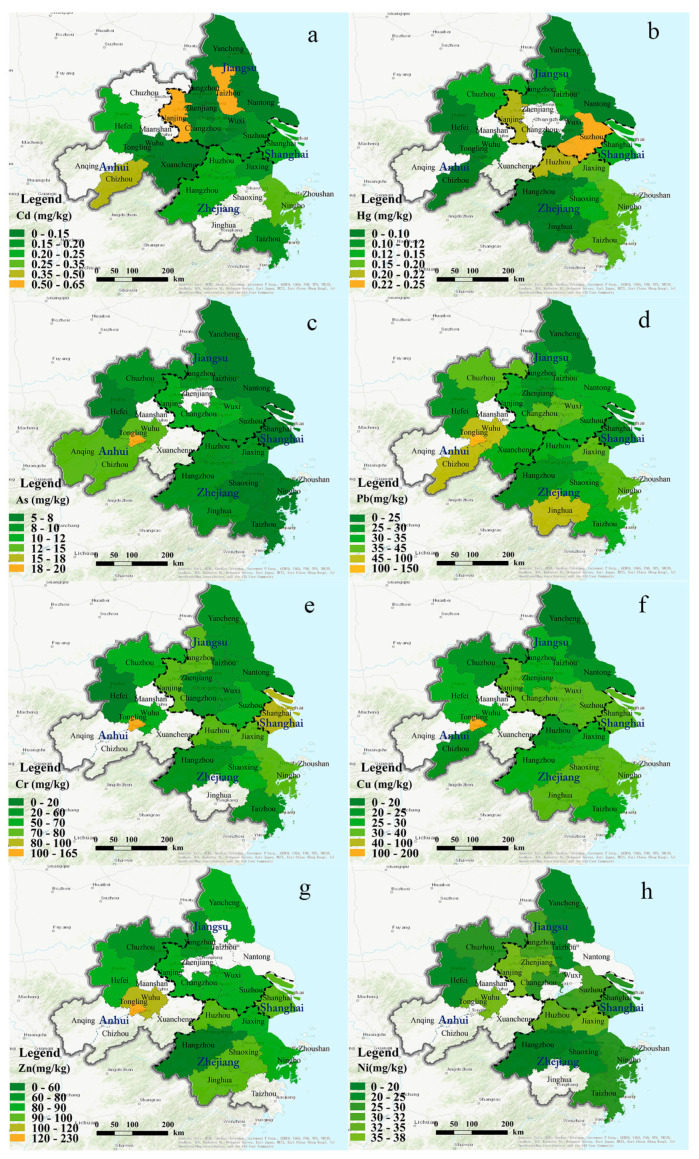
Spatial distribution of (**a**) Cd; (**b**) Hg; (**c**) As; (**d**) Pb; (**e**) Cr; (**f**) Cu; (**g**) Zn; and (**h**) Ni concentrations in the cities of the Yangtze River Delta (YRD).

The article numbers were counted and illustrated by points that differ in size based on county-level division (Figure 2). We found that more researchers were concerned about the soil environment in regions along the eastern seaboard, such as Shanghai, Ningbo (Zhejiang), and Suzhou (Jiangsu), and what they have in common are frequent industrial activities and high population density. In addition to the overall pollution situation analysis of the YRD, the spatial distribution was analysed by the administrative division. The specific calculated mean concentrations of eight elements in the soil were performed in Table S1. Figure 3 shows the results of the average concentration calculated by provinces in the YRD, including Shanghai, Jiangsu, Zhejiang, and Anhui, as compared with the background values. There was no obvious PTEs accumulation in any province’s agricultural soil, with the exception of Cd and Hg. The Cd concentration in Anhui was significantly higher than its background value, as was the Hg content in Jiangsu. Moreover, a subgroup analysis based on the city level was conducted, wherein darker colours represent higher concentrations (Figure 4).

The Cd concentrations in Chizhou (Anhui), Nanjing (Jiangsu), and Ningbo (Zhejiang) were higher than those of other cities, while the Hg and Pb concentrations were higher in Nanjing (Jiangsu) and Wuhu (Anhui). Note that the Pb, Cr, Cu, and Zn contents in Tongling (Anhui) were the highest in the entire YRD. The main reason contributing to this is that Tongling is famous as an important mining region in China which may lead to clear accumulation of soil potentially toxic elements.

### 3.4. Ecological Risk Assessment of PTEs in the YRD

**Figure 5:**
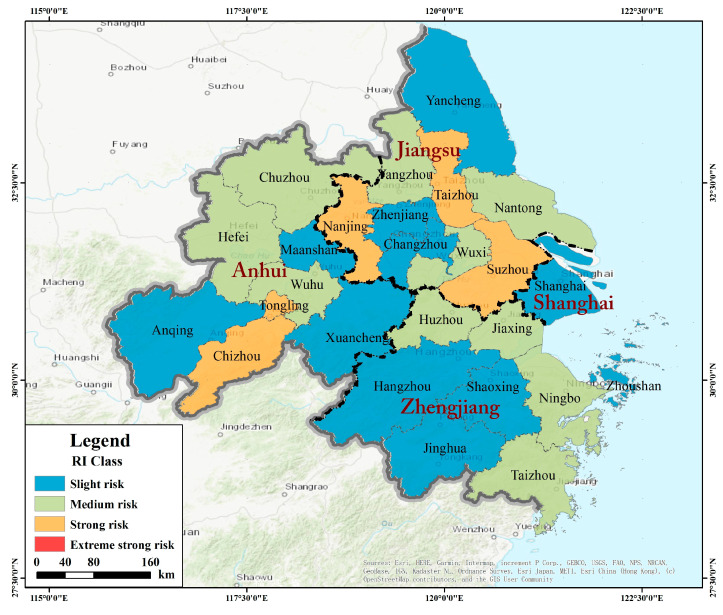
Ecological risk levels of potentially toxic elements (PTEs) in the cities of the Yangtze River Delta (YRD).

The ecological risks of each province and the entire YRD were assessed using Hakanson’s ecological risk index [32] (Table 2). Hakanson gave the corresponding risk index classification standard (RI < 150 is low, 150 ≤ RI < 300 is moderate, 300 ≤ RI < 600 is considerable, and RI ≥ 600 is very high). The ecological risk level of PTEs in the agricultural soil of the entire YRD was moderate, wherein the risk was predominantly contributed by Hg and Cd, while other PTEs s did not pose significant ecological risks. The PTEs pollution status of the four provinces showed a trend of Jiangsu > Anhui > Zhejiang > Shanghai, and the risk levels of them were considerable, considerable, moderate, and low. The major pollutant PTEs was Hg in the agricultural soil of Jiangsu, and Cd in that of Anhui. Ecological risk assessments and mapping were conducted for 25 cities in the YRD (Table 2, Figure 5). In terms of cities, no city among them reached a ‘very high’ ecological risk level, 10 cities were of moderate risk, 10 were of low risk, and five were of considerable risk, and the main contaminations in considerable risk cities were Cd and Hg. The results revealed that Tongling (Anhui), Nanjing (Jiangsu), Taizhou (Jiangsu), Suzhou (Jiangsu), and Chizhou (Anhui) are facing a serious pollution risk, wherein Tongling, Chizhou, and Taizhou were mainly contaminated by Cd, while Nanjing, and Suzhou were primarily contaminated by Hg.

### 3.5. Temporal Trend Analysis of PTEs in Agricultural Soil of the YRD

**Figure 6:**
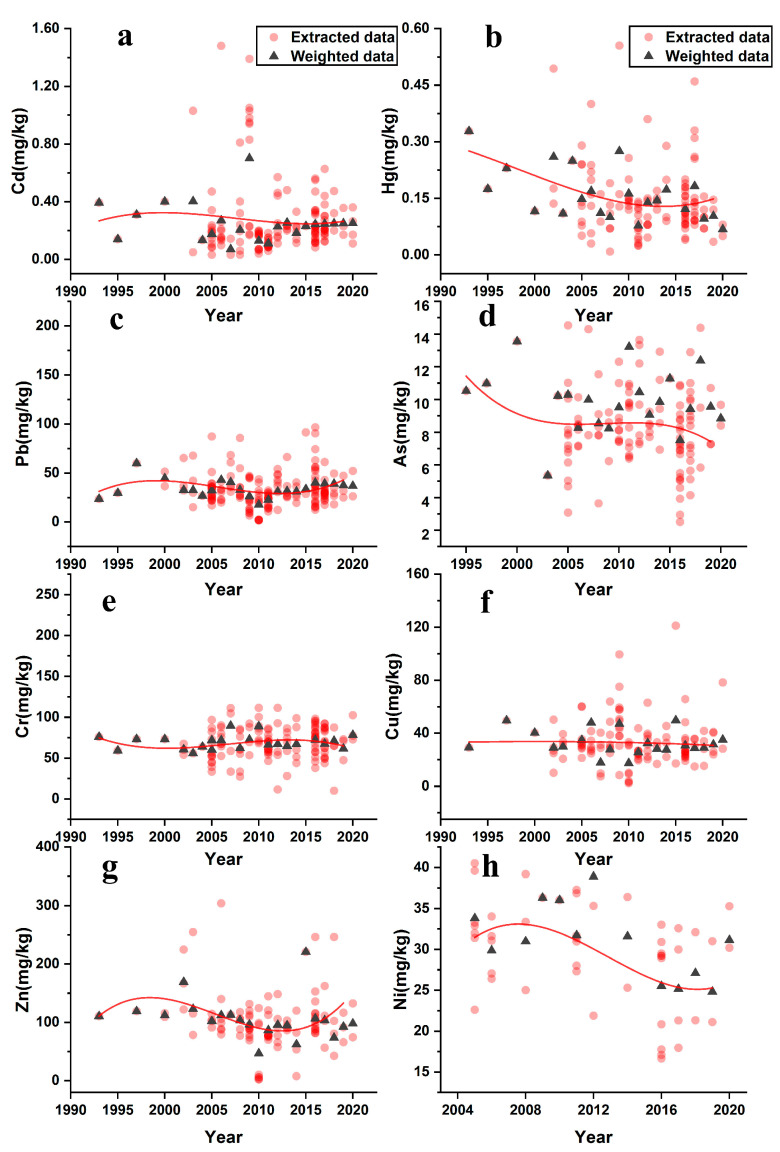
Temporal trend of (**a**) Cd; (**b**) Hg; (**c**) As; (**d**) Pb; (**e**) Cr; (**f**) Cu; (**g**) Zn; and (**h**) Ni contents in agricultural soil in the Yangtze River Delta (YRD); (red points represent all data extracted from articles except outliers; black triangles represent the weighted mean data grouped by years; and red curve is a nonlinear fitting for the extracted data).

To further understand the change mechanism and trend of PTEs pollution in the agricultural soil of the YRD, the literature databases were grouped and calculated for a single year from 1993 to 2020. All the data extracted from the literature and weighted mean values of a single year are shown in Figure 6, wherein the temporal variation is represented by a polynomial regression curve. With the change of time, the contents of eight PTEs in the soil have different trends. Compared with other elements, the fluctuations of Cu and Cr contents were slight. The contents of Cd, Pb, and Zn in the agricultural soil showed an overall increasing trend from 1993 to 2000 and then showed a decreasing trend. The Hg, As, and Ni concentrations in the agricultural soil of the YRD showed continuous decreasing trends of different rates for different periods.

## 4. Discussion

### 4.1. PTEs Pollution Characteristics in the YRD

The weighted mean value calculated by the meta-analysis showed that soil PTEs pollution in the YRD was generally slight. However, Cd and Hg presented a relatively stronger risk of pollution due to their higher accumulation and toxicity levels, especially in some cities in eastern YRD, such as Suzhou (Jiangsu), Nanjing (Jiangsu), and Ningbo (Zhejiang). Previous studies found that human activities, such as industry, agriculture, and transportation, have significant impacts on the soil environment, playing a critical role in the processes of accumulation, spatial distribution, and migration of PTEs in the soil [42,43]. In industrial production processes, wastewater, waste gas, and waste residue directly or indirectly pollute the soil environment [44,45]. In Suzhou, the atmospheric deposition was proved the most important source of soil Hg [46]. The main anthropogenic source of atmospheric mercury involves coal-fired power plants and industrial furnaces in the YRD [47]. As one of the world’s largest industrial cities, Suzhou has numerous industrial boiler equipment and large coal-fired power plants, which could be the cause of its serious soil Hg pollution. Researchers analysed the source in an abnormally high soil heavy metals agricultural area in Nanjing and found that the annual input flux of Cd in the soil through atmospheric deposition reached 7.00 g hm^−2^, which may be mainly related to chemical activities such as coking in industrial parks near the study area. At the same time, agricultural fertilisation is also an important source; the annual input flux of Cd in the soil via agricultural fertilisation reached as high as 8.94 g hm^−2^ [48]. In addition, PTEs with high availability are released into the environment directly during the process of metal mining, especially for copper mines [49,50]. For this reason, strong risk of PTE pollution was present in Tongling, which was engaged in frequent copper mining for decades.

### 4.2. Temporal Variation of Soil PTEs Content in YRD

As previously mentioned, the concentration of PTEs, such as Cd, Pb, and Zn, first increased and then decreased with an inflection point around 2000. According to the mass balance theory, when the input is greater than the output, PTEs accumulate in the soil; otherwise, the opposite occurs [51]. Numerous studies have shown that fertiliser and pesticide applications are directly related to PTEs accumulation, such as Cd, Pb, and Zn, in agricultural soil [52,53,54,55]. To relieve environmental pressure, regulations have been implemented to limit the use of fertilisers and pesticides, including prohibiting pesticides containing Hg, As, and Pb in Chinese agriculture since 2002 [56]. Meanwhile, industrial emissions were also an important source of soil PTEs pollution. Since the 1990s, with the Chinese economy reforming and opening-up, the industry in the YRD region entered a stage of accelerated development. Therefore, the PTEs concentration in agricultural soil in the YRD was higher in the early 21st century. Recently, to establish a sustainable economic development model, the Chinese government adopted a series of policy reforms and control measures to alleviate soil PTEs pollution [57].

The output of PTEs in agricultural soil main through crop removal, leaching, and surface runoff [58], and the proportion of their output contribution varies in different regions. In Zhejiang, researchers proved that the annual flux of Cd output from farmland through crop harvest and leaching were 1.26 g hm^-2^ and 1.80 g hm^-2^, contributing 34.52% and 49.32% of the total output flux, respectively [59]. However, researches usually proved the leaching losses and crop uptake were usually relatively small compared with the total fluxes of PTEs input into the agricultural soil [60,61]. Therefore, the decrease of PTEs contents in the soil is more likely be the result of the anthropogenic intervention that is the remediation and treatment of contaminated soil. Phytoextraction of Zn and Cd contaminated soil by hyperaccumulator, e.g., Sedum plumbizincicola, has been shown to be effective in the YRD [62]. However, as soil PTEs pollution is highly hazardous, long-term, and irreversible, soil pollution prevention and control should be an ongoing effort.

### 4.3. Comparison with Previous Studies

Comparisons between the agricultural soil PTEs content in the YRD and other regions in China, including the Pearl River Delta (PRD), Huabei Plain, and other provinces [63,64,65,66,67,68,69], are summarised in Table 3. The results of this study were highly consistent with the field monitoring results of the YRD farmland soil by Shao et al. [26], especially for Cd, Pb, and Ni. However, the concentrations of Cu and Zn in this study were slightly higher than those found by Shao et al. This discrepancy is probably due to the existence of publication bias, as the concentrations in the ‘normal group’ of Cu (28.69 mg kg^−1^) and Zn (90.61 mg kg^−1^) were closer to the field measured results in the YRD. Meanwhile, our results were consistent with the national field monitoring results of Song et al. [70], indicating that PTE pollution in the YRD was at the national average level. The results of this study were also compared with those of the Hunan Province, whose PTE pollution situation has attracted widespread attention as a result of public events, including ‘cadmium rice’ [71,72]. Apparently, the concentrations of PTEs in the soil of the YRD were lower than those in the Hunan Province, especially for Cd, As, and Zn (Table 3). In addition, as an economically developed region, the PRD showed more serious soil PTE contamination than the YRD. However, compared with southern China (YRD, PRD, and Fujian), the soil in northern China (Heilongjiang and Hebei) had less PTE pollution [53]. In summary, the results of the meta-analysis were confirmed to be reliable, revealing that the PTE pollution level of agricultural soil in the YRD was close to the national average level. However, PTE pollution in the agricultural soil of the YRD should not be underestimated, especially for Cd and Hg, even though the pollution levels in the YRD were relatively low compared with other regions such as Hunan Province and the PRD.

**Table 3:** Average contents of soil potentially toxic elements (PTEs) in the soil of various Chinese regions (mg kg^−^^1^).

Region	Cd	Hg	As	Pb	Cr	Cu	Zn	Ni	Field Sampling/Review	Reference
YRD, China	0.25	0.14	8.14	32.32	68.84	32.58	92.35	29.30	Review (118 articles)	This study
YRD, China	0.23			37.63		25.82	88.38	29.21	240 soil samples	[26]
Pearl River Delta, China	0.58			40.00	71.40	33.00	84.70	21.10	38 soil samples	[63]
Heilongjiang, China	0.10	0.05	8.53	21.29	59.45			26.04	450 soil samples	[65]
Hebei, China	0.15	0.08	6.16	18.80	57.77	21.22	69.96	25.04	100 soil samples	[66]
Shanxi, China		0.61	10.72		76.69	30.19	87.69	43.87	126 soil samples	[64]
Fujian, China	0.26		22.60	91.94	49.01	27.92		27.46	272 soil samples	[67]
Hunan, China	0.85	0.25	21.05	56.06	74.96	38.85	147.28	26.83	Review	[68]
China	0.25	0.16	9.50	34.90	65.30	30.70	85.30	30.70	138 soil samples	[69]

## 5. Conclusions

This paper reviewed 118 studies about agricultural soil PTEs contamination published between 1993 and 2020 in China. Overall, the concentration of PTEs did not exceed the national standard but was close to the national average level. With the exceptions of Cd and Hg, the PTEs did not show significant accumulation in the soil as compared with the background value. The eastern YRD showed a higher risk of pollution due to the industrial agglomeration effect, wherein some cities, such as Tongling (Anhui), had a higher risk of PTEs pollution due to robust mining activities. At the beginning of the 21st century, the PTEs content in soil was relatively higher and then experienced a decreasing trend. The available data provide a reference for the prevention, treatment, and remediation of soil pollution by PTEs in the YRD, by the environmental agencies.

## Figures and Tables

**Table 1:** Regional mean values (mg kg^−1^) of eight elements in agricultural soil in the Yangtze River Delta (YRD).

Element	Cd	Hg	As	Pb	Cr	Cu	Zn	Ni
Numbers of sites	153	121	127	175	137	120	104	49
Number of sampling points	27,210	255,447	29,290	31,432	22,183	20,888	20,104	10,549
Weighted mean values ^a^	0.25	0.14	8.14	32.32	68.84	32.58	92.35	29.30
Number of outliers	2	1	1	0	2	0	1	0
Background values ^b^	0.10	0.07	11.20	26.00	61.00	22.60	74.20	26.90
Standard values ^c^	0.30	0.50	30.00	80.00	250.00	150.00	200.00	60.00
Percentage of exceedances ^d^	26.14%	1.65%	1.57%	3.43%	0	0.83%	6.94%	0

^a^ Regional weighted mean values were calculated using the database without outliers. ^b^ Background values [37]. ^c^ Standard values were derived from national specification (GB 15618-2018) elements screening value (paddy fields with pH ≤ 5.5). ^d^ Percentage of sites exceeding the standard values (GB 15618-2018).

**Table 2:** The regional ecological risk index (RI) of eight elements in agricultural soil in the Yangtze River Delta (YRD).

Region	City	Cd	Hg	As	Pb	Cr	Cu	Zn	Ni	Total
Entire YRD		77.3	88.6	7.3	6.2	2.2	7.2	1.2	5.4	195.5
Shanghai		36.9	43.2	10.3	6.6	2.9	6.1	1.2	4.3	111.6
Jiangsu		97.1	264.9	9.1	7.0	1.6	7.0	1.3	4.5	392.6
	Nanjing	170.7	269.0	11.8	7.2	2.0	7.9	1.5	5.2	475.3
	Wuxi	49.2	121.4	12.0	9.1	1.5	7.2	1.3	0.0	201.8
	Changzhou	62.0	0.0	11.2	8.3	1.4	7.9	1.5	4.3	96.6
	Suzhou	55.7	297.3	9.7	7.4	1.7	7.2	1.4	4.5	384.9
	Nantong	23.2	115.7	8.0	6.9	1.1	4.3	0.0	0.0	159.2
	Yancheng	36.5	52.0	7.7	3.4	1.6	3.8	1.4	3.5	109.8
	Yangzhou	44.0	138.7	10.5	5.7	1.9	5.8	1.1	4.6	212.3
	Zhenjiang	42.0	0.0	0.0	5.7	1.5	6.0	0.0	5.3	60.5
	Taizhou	206.7	164.0	7.8	6.9	1.5	5.6	0.0	3.6	396.1
Zhejiang		45.7	128.7	7.6	6.1	2.1	4.9	1.1	5.4	201.6
	Hangzhou	39.7	62.7	8.7	4.5	1.4	5.0	0.5	3.4	12.9
	Ningbo	55.7	135.6	5.6	7.3	2.3	6.1	1.0	5.7	219.4
	Jiaxing	35.0	156.5	9.5	6.5	2.1	4.7	1.0	6.9	222.0
	Huzhou	44.0	159.8	9.0	5.0	2.1	3.9	1.1	6.6	231.6
	Shaoxing	0.0	103.5	7.8	5.7	1.9	5.3	1.1	4.5	129.8
	Jinhua	0.0	56.2	9.6	8.0	0.0	6.5	1.2	0.0	81.6
	Taizhou	28.7	148.2	7.8	5.1	1.7	4.6	0.0	4.8	200.9
Anhui		181.9	111.3	10.6	9.5	2.6	6.2	1.6	4.6	328.4
	Hefei	120.0	46.7	4.9	5.8	0.8	4.7	1.4	3.1	187.3
	Wuhu	13.0	154.7	11.3	11.4	2.6	4.7	1.9	5.4	205.0
	Maanshan	0.0	16.7	0.0	0.0	0.0	0.0	0.0	0.0	16.7
	Tongling	420.0	108.4	17.2	32.1	6.4	34.4	3.8	0.0	513.9
	Anqing	0.0	0.0	11.6	0.0	0.0	0.0	0.0	0.0	11.6
	Chuzhou	0.0	185.3	7.1	8.8	2.7	4.0	1.0	4.2	213.1
	Chizhou	236.7	107.5	10.5	10.4	0.0	4.3	0.0	0.0	369.3
	Xuancheng	62.0	0.0	0.0	7.4	0.0	0.0	0.0	0.0	69.4

The national eight elements background values were adopted in the RI calculation of the entire YRD, and the regional background values were used for the provinces and cities.

## Supplementary Materials

The following are available online at https://www.mdpi.com/1660-4601/18/3/1033/s1. Figure S1: Probability distribution histogram of eight elements extracted from all studies. Table S1: The calculated concentration of eight elements in agricultural soil in the Yangtze River Delta (YRD) (mg kg-1).
