# Supplementary material for: Current Status and Temporal Trend of Potentially Toxic Elements Pollution in Agricultural Soil in the Yangtze River Delta Region: A Meta-Analysis
Source: Int J Environ Res Public Health. 2021 Jan 25;18(3):1033. doi: 10.3390/ijerph18031033 (PMC7908581; doi:10.3390/ijerph18031033)
Supplement: Supplementary file 1 [file ijerph-18-01033-s001.zip › supplementary/supplementary 1.docx]

**Supporting Information**

Current status and temporal trend of potentially toxic elements pollution in agricultural soil in the Yangtze River Delta Region: A meta-analysis

Shufeng She ^1^, Bifeng Hu ^1,2,3^, Xianglin Zhang ^1^, Shuai Shao ^1^, Yefeng Jiang ^1^, Lianqing Zhou ^1^ and Zhou Shi ^1,^*

^1^ Institute of Applied Remote Sensing and Information Technology, Zhejiang University,
Hangzhou 310058, China; 21814120@zju.edu.cn (S.S.); hubifeng@zju.edu.cn (B.H.); zhangxianglin@zju.edu.cn (X.Z.); sshuai@zju.edu.cn (S.S.); [jiangyefeng@zju.edu.cn](mailto:jiangyefeng@zju.edu.cn) (Y.J.); [LianQing@zju.edu.cn](mailto:LianQing@zju.edu.cn) ( L.Z.);

^2^ Department of Land Resource Management, School of Tourism and Urban Management, Jiangxi University of Finance and Economics, Nanchang 330013, China.

^3^ Unité de Recherche en Science du Sol, INRAE, Orléans, 45075, France.

***** Correspondence: [shizhou@zju.edu.cn](mailto:shizhou@zju.edu.cn); Tel.: +86-571-8898-2831


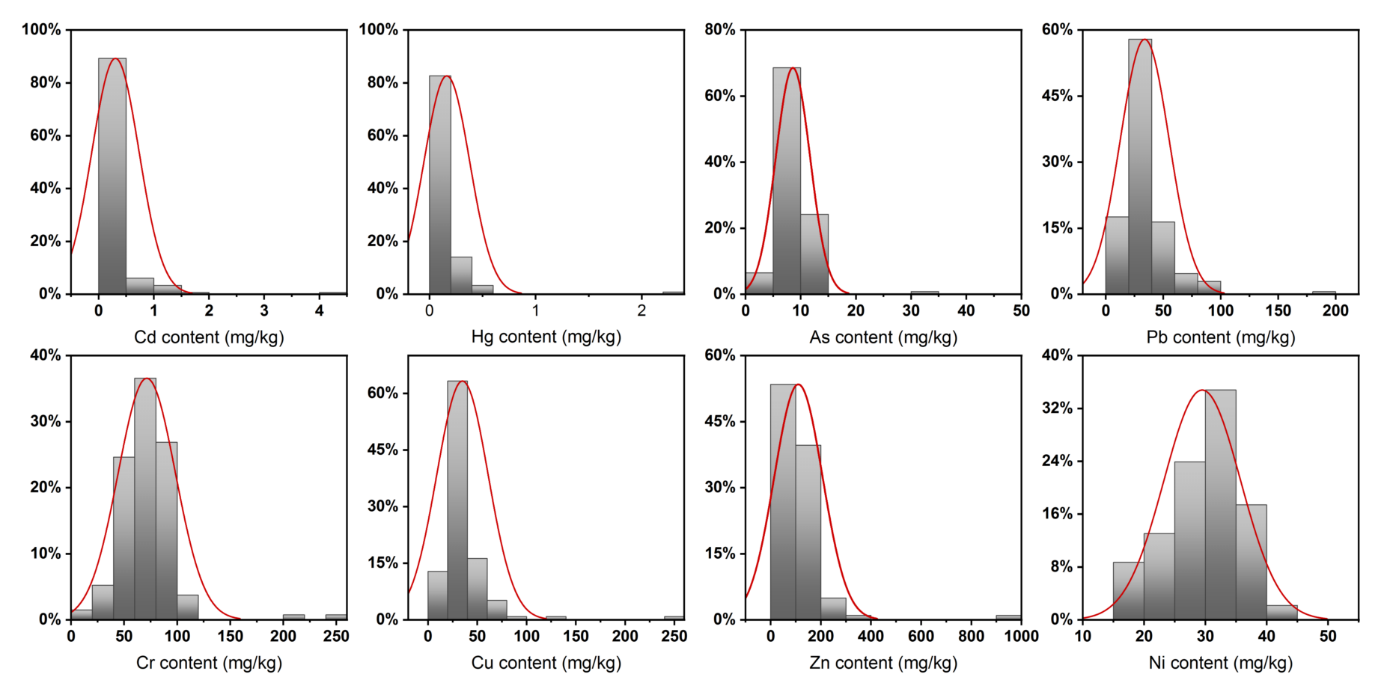


**Figure S1.** Probability distribution histogram of eight elements extracted from all studies.

| **Region** |  | **Cd** | **Hg** | **As** | **Pb** | **Cr** | **Cu** | **Zn** | **Ni** |
| --- | --- | --- | --- | --- | --- | --- | --- | --- | --- |
| **All over YRD** | | **0.25** | **0.14** | **8.14** | **32.32** | **68.84** | **32.58** | **92.35** | **29.30** |
| **Shanghai** |  | **0.21** | **0.14** | **7.80** | **29.69** | **75.33** | **33.29** | **108.95** | **26.81** |
| **Jiangsu** |  | **0.29** | **0.20** | **8.31** | **31.62** | **61.98** | **33.44** | **86.52** | **30.59** |
|  | Nanjing | 0.51 | 0.20 | 10.73 | 32.71 | 74.14 | 37.85 | 98.11 | 35.32 |
|  | Wuxi | 0.15 | 0.09 | 10.92 | 41.26 | 58.60 | 34.58 | 86.25 | 0.00 |
|  | Changzhou | 0.19 | 0.00 | 10.17 | 37.54 | 54.61 | 38.01 | 96.94 | 29.24 |
|  | Suzhou | 0.17 | 0.22 | 8.81 | 33.32 | 66.15 | 34.80 | 89.14 | 30.33 |
|  | Nantong | 0.07 | 0.09 | 7.25 | 31.17 | 40.96 | 20.70 | 0.00 | 0.00 |
|  | Yancheng | 0.11 | 0.04 | 7.05 | 15.32 | 59.13 | 18.25 | 89.56 | 23.33 |
|  | Yangzhou | 0.13 | 0.10 | 9.53 | 25.78 | 72.21 | 27.71 | 74.24 | 31.20 |
|  | Zhenjiang | 0.13 | 0.00 | 0.00 | 25.95 | 55.75 | 28.78 | 0.00 | 35.76 |
|  | Tazhou | 0.62 | 0.12 | 7.14 | 31.12 | 56.26 | 26.90 | 0.00 | 24.08 |
| **Zhejiang** |  | **0.26** | **0.15** | **7.11** | **37.22** | **72.82** | **28.61** | **100.12** | **28.57** |
|  | Hangzhou | 0.22 | 0.08 | 8.09 | 27.65 | 47.90 | 29.03 | 47.96 | 17.96 |
|  | Ningbo | 0.31 | 0.17 | 5.23 | 44.47 | 79.54 | 35.50 | 94.29 | 29.99 |
|  | Jiaxing | 0.19 | 0.20 | 8.83 | 39.33 | 70.61 | 27.21 | 91.61 | 36.19 |
|  | Huzhou | 0.25 | 0.20 | 8.39 | 30.26 | 71.69 | 22.97 | 102.20 | 34.66 |
|  | Shaoxing | 0.00 | 0.13 | 7.27 | 34.41 | 65.06 | 31.15 | 103.13 | 23.82 |
|  | Jinhua | 0.00 | 0.07 | 8.93 | 48.80 | 0.00 | 38.22 | 105.75 | 0.00 |
|  | Zhoushan | 0.00 | 0.00 | 0.00 | 0.00 | 0.00 | 0.00 | 0.00 | 0.00 |
|  | Taizhou | 0.16 | 0.19 | 7.27 | 30.91 | 57.74 | 26.90 | 0.00 | 25.01 |
| **Anhui** |  | **0.35** | **0.08** | **12.72** | **42.12** | **66.48** | **33.81** | **96.42** | **30.18** |
|  | Hefei | 0.24 | 0.04 | 5.83 | 25.95 | 19.86 | 26.14 | 86.45 | 21.33 |
|  | Wuhu | 0.03 | 0.12 | 13.50 | 51.35 | 67.83 | 26.46 | 115.50 | 36.87 |
|  | Maanshan | 0.00 | 0.01 | 0.00 | 0.00 | 0.00 | 0.00 | 0.00 | 0.00 |
|  | Tongling | 0.84 | 0.08 | 20.63 | 144.68 | 163.62 | 193.29 | 225.32 | 0.00 |
|  | Anqin | 0.00 | 0.00 | 13.87 | 0.00 | 0.00 | 0.00 | 0.00 | 0.00 |
|  | Chuzhou | 0.00 | 0.14 | 8.52 | 39.69 | 69.07 | 22.36 | 60.53 | 28.44 |
|  | Chizhou | 0.47 | 0.08 | 12.55 | 46.77 | 0.00 | 24.05 | 0.00 | 0.00 |
|  | Xuancheng | 0.12 | 0.00 | 0.00 | 33.10 | 0.00 | 0.00 | 0.00 | 0.00 |

**Table S1.** The calculated concentration of eight elements in agricultural soil in the Yangtze River Delta (YRD)( mg kg^-1^).
